# Thermo-mechanical properties of pretreated coir fiber and fibrous chips reinforced multilayered composites

**DOI:** 10.1038/s41598-021-83140-0

**Published:** 2021-02-11

**Authors:** K. M. Faridul Hasan, Péter György Horváth, Zsófia Kóczán, Tibor Alpár

**Affiliations:** 1 Simonyi Károly Faculty of Engineering, University of Sopron, Sopron, Hungary; 2 Simonyi Károly Faculty of Engineering, Paper Research Institute, University of Sopron, Sopron, Hungary

**Keywords:** Plant sciences, Materials science

## Abstract

Coir is one of the most important natural fibers having significant potentiality in structural biocomposites production. The long coir fiber (LCF) and short fibrous chips (CFC) were extracted from the husk of coconut. The dimensions of the CFC were within 1.0–12.5 mm and the LCF were within 2.0 mm. All the fibers and fibrous chips were treated with 5% NaOH (alkali) before the biocomposite manufacturing. Different percentages (8%, 10%, and 12%) of melamine-urea-formaldehyde (MUF) were used to produce the tri-layered medium density composite panels with 12 mm thickness. The mechanical properties (tensile, flexural, and internal bonding strengths) of coir reinforced multilayered composites has been studied for all the produced biocomposites. The morphological, micro-structural, and bonding mechanisms were investigated by Scanning electron microscope and Fourier-transform infrared spectroscopy analysis. Thermal properties of the biocomposites were studied by thermal conductivity, thermogravimetric analysis, and derivative thermogravimetry characterization. The moisture contents of the final composite panels were also investigated in this study. The main objective of this work is to investigate the influences of MUF on treated coir fiber and fibrous chips reinforced tri-layered biocomposites. Beside, a novel sustainable product is developed through reinforcing the fibrous chip with coir fiber in terms of multilayered biocomposite panels.

## Introduction

Natural fibers have become prominent reinforcement materials in terms of sustainable, biodegradable^1–3^, non-toxic, and environment-friendly^4^ features especially for biocomposites production. Besides, natural fibers also minimize the emissions of CO_2_ to the surrounding environment. Biocomposites are getting popularity as attractive products in automotives, aeronautical, packaging, construction, building, biomedical, and so on^2,5–7^. Furthermore, natural fibers are available throughout the globe which are cheaper, providing higher stiffness and recyclable property in contrast to the artificial fibers^8–12^. Coir is one of the prominent natural fiber considered for higher strength and durable product material, which are collected from the ripe coconut fruits husk^13,14^. Coir is processed from the husk of coconut fruits which are widely available in some tropical regions such as Vietnam, Thailand, Sri Lanka, Bangladesh, India, Brazil, Indonesia, and so on. Annually, 42 million metric tons of coconuts are produced around the world. The coconut husk encompasses nearly 75% fibers and another 25% so called fine “coir pith”^15^. Furthermore, coir fibers are also comprised with 36‒43% cellulose, 32.25% lignin, and 15.17% hemicellulose like as many other naturally originated fibers^16^. The most important fact for coir is the lower degradation rate because of having higher lignin content^17,18^. Coir fibers have some competitive advantages over the other natural fibers like low cost, low density, higher elongation at break, and lower elastic modulus^19,20^. Furthermore, coir fiber generally exists 1.1 to 1.5 g/cm^3^ density, 105 to 593 MPa tensile strength, and 2 to 8 GPa Youngs modulus^20^.

Previously, coir fibers were used for geotextiles (biodegradable fabrics), which were mainly used for erosion control from rain or dams of rivers and beaches^21^. However, processed coir from ripe coconuts were also used for automotives especially as the base material of seats^22^. These materials need to be recycled after the end of service life instead of burning for the sustainable disposals to reduce burdens from the environment. Besides, the recycling of automotive parts are also significantly important according to the European standard, 2000/53 ELV- “End of Life Vehicles” regulations (article 7)^22^. The vehicle companies are also putting efforts on utilizing biodegradable products to avoid burning processes through quick recovery/recycling after the end use. There are different processing methods available for producing coir fiber reinforced biocomposites like open molding, compression molding, resin transfer molding (RTM), injection molding, extrusion, and so on^20^. However, compression molding is a popular method as it could process high volume of fiber at high temperature and pressures^23^. The implementation of compression molding possesses some benefits over the other methods in terms of economical perspective, low production volume, short production cycle, better dimensional stability, uniform density, and better thermo-mechanical properties.

Coir fiber-based biocomposites exhibit lower tensile strength and stiffness for the presence of lower amount of cellulose and hemicellulose^24^. Besides, the impurities are also responsible to affect the better interactions between the polymer and fibers in the matrix^2^. There were also several studies highlighted about the pretreatment of the coir before the biocomposite productions^20,25–27^. The cellulosic and hemicellulosic groups of natural lignocellulosic fibers bear polarized ‒OH (hydroxyl) groups^28^. Besides, they are also hydrophilic fibers which limits industrial applications for absorbing moisture from the surrounding atmosphere, thus provides weaker interfacial bond between the natural fiber and polymeric matrix^20^. However, the pretreatment of natural fibers could improve this problem through enhancing interfacial adhesion between them. The most commonly used treatment methods are silane modification, alkali treatment, mercerization, enzymatic treatment, corona, and plasma treatments^29,30^. However, the alkaline treatments of natural fiber is one of the most efficient methods used for fiber treatment^20,31,32^. It removes wax, oil, and different impurities present in the fiber. Conversely, it also increases the surface roughness thus yields mechanical features of the biocomposites. Beside, alkaline treatment also ensures the better wettability of natural fibers. The reaction mechanism between coir and NaOH is given in Eq. (1). Where, long coir fibers are indicated by LCF and coir fibrous chips by CFC.

1 $${\text{Coir }}\left( {{\text{LCF}}/{\text{CFC}}} \right) - {\text{OH}} + {\text{NaOH}} \to {\text{Coir }}\left( {{\text{LCF}}/{\text{CFC}}} \right) - {\text{O}}^{ - } - {\text{Na}}^{ + } + {\text{H}}_{2} {\text{O}}$$

So, both types of coir fibers (LCF and CFCs) were treated with alkaline solutions of NaOH. Besides, MUF is a strong binding agent that could be used for biocomposites production as it has very good interaction with the natural fibers compared to other polymeric resins^33^. MUF is a white, odorless, and tasteless binding adhesive. MUF is also an amino resin which possesses the combined advantages of other two types of formaldehyde resins (urea formaldehyde and melamine urea formaldehyde)^34^. The main chemical constituents of MUF resins are N‒H, O‒H, C‒H, C═O, and C═ONH_2_^34–37^. However, the pH, density, solid content, and viscosity are some of the influential and prominent quality parameters of industrially usable MUF resins^34^. Recently, different particle boards, laminates, and water resistant composite manufacturing companies are using the MUF resin commercially. Besides, the MUF polymeric resin used in case of woods provide higher Youngs modulus compared to the other polymeric resins like polyester^33^. The MUF polymeric resin has very good capability for successful attachments at the surface of cellulosic material, E-glass fibers, CaCO_3_, and so on^38^. However, the biocomposites made of multilayered CFC and LCF materials is not yet researched which could have significant potentiality to produce composite panels commercially. The tri-layered coir-based biocomposites were developed by placing CFCs as the core layer and LCFs of different proportions as the upper and bottom layers. According to our knowledge, this is the first time we are going to report pretreated CFC (fiber chips even at 12.5 mm range of dimensions) and LCF reinforced biocomposites through using MUF polymeric adhesive.

## Experimental

### Materials

Coir materials (LCF and CFC) (*cocos nucifera*) were kindly donated by Pro Horto Ltd., a well-known company located in central Europe (Szentes, Hungary) for the purpose of research. The chemical components of coir materials are provided in Table 1. Alkaline NaOH, CAS-No. 1310-73-2, was purchased from VWR international Kft., (Debrecen, Hungary). The MUF (KRONORES MD 2141 J) was supplied by SC Kronospan Sebes SA, Romania. The solid content of the MUF was 63 ± 2%, density 1.25‒1.28 g/cm^3^, and viscosity 100‒250 mPas with white milky color appearances in liquid form.

**Table 1:** Chemical composition of coir.

Chemical compound	Content (%) by AlNous et al.^15^	Content (%) by Kochova et al.^39^
Cellulose	26.8 ± 0.05	36.6
Lignin	30.5 ± 0.02	22.2
Hemicellulose	17.2 ± 0.05	37.0
Extractives	22.01 ± 0.02	4.2
Ash	3.7	1.9
Total	100.21	101.9

### Preparation and pretreatment of coir

Initially, the LCFs were cut around 2 mm length through ensuring homogeneous diameter. The CFCs were sieved to ensure uniform dimensions by a Sieve analyzer (Fritsch GmbH, ANALYSETTE 3 Pro, Weimar, Germany) with different DIN sieves ranging within 1.0 to 12.5 mm. The maximum fiber dimensions were between 4.5 and 12.5 mm range as shown in Fig. 2a. However, there is also significant presence of fibers in other ranges as well (Fig. 2a). The sieve analysis was performed for 100 g samples with 2.0 amplitude vibrations for 15 min. Both the LCFs and CFCs were treated with 5% (w/v) NaOH at alkaline conditions (pH around 12) for 24 h at environmental temperatures to increase the interfacial adhesions between the coir materials and MUF polymer matrix. The LCFs and CFCs were then washed with cold water for three times after 30 min of immersion into the water to remove the cleaned impurities and mucus of NaOH from the fiber surfaces. The fibers were then air dried under sunlight (temperature around 20‒22 °C) for two days at summer season in Sopron, Hungary. The moisture content for both LCF and CFCs were measured by using Kern ULB 50-3 N made by KERN AND SOHN GmbH (Germany). The average moisture content for both types of coir was 7.3 ± 0.3%. The accuracy of the balance during the measurement was 0.001 g and temperature 105 ± 0.3 °C. The standard adopted for moisture content analysis of coconut materials were EN 322:1993.

**Figure 1:**
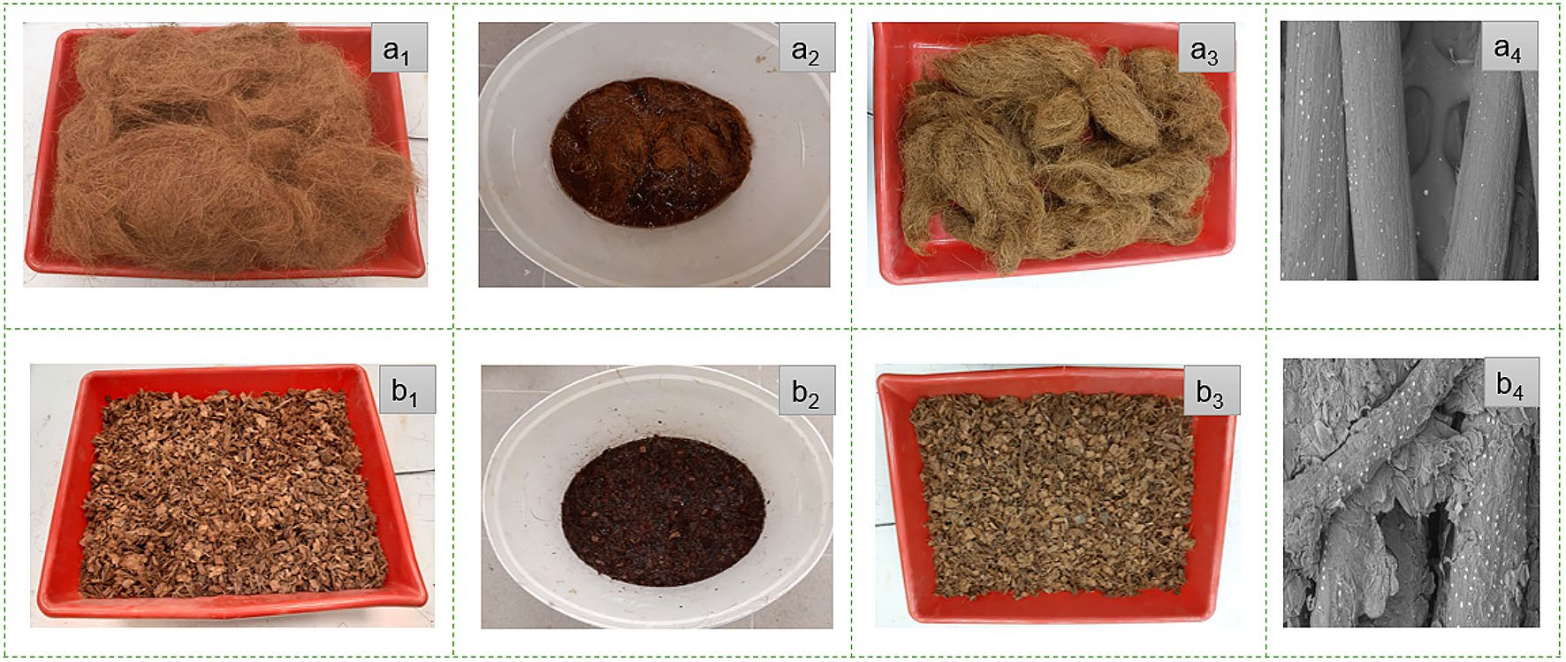
Photographs of coir fibers: (a_1_ and b_1_) before pretreatment; (a_2_ and b_2_) coir fibers in alkaline solutions; (a_3_ and b_3_) coir fibers after treatment; (a_4_ and b_4_) morphological views of coir fibers**.**

The pretreatment of the coir by alkaline solution helps to remove the impurities present in the fiber surface. The photographs of untreated, during the treatment, and after the treatment of coir (both LCF and CFCs) are shown in Fig. 1. As the coir contains low cellulose, so the Na^+^ of caustic soda could easily be diffused into the coir structure as the polymeric solvents could easily get access into the amorphous region of natural fibers^40^. However, the washing of coir also removes the Na^+^ from the crystallization region of fibers^41,42^. However, the usage of higher concentration of NaOH could degrade the coir fibers cell ^43,44^, so it is tried to use the optimized alkali concentration (5%) to treat the coir fibers with traditional mercerization method^2,45,46^ for avoiding any damage of cellulosic structures. So, the composites developed from the treated fibers provided better mechanical properties as shown in Table 2.

**Table 2:** Different recipes of coir fiber reinforced MUF polymeric biocomposites (C@BC 1, C@BC 2, and C@BC 3).

Chemical/Materials	U‒LCF (%)	C‒CFC (%)	L‒LCF (%)	MUF (%)	H (%)
C@BC 1	3.58	82.85	3.58	8	2
C@BC 2	3.57	80.86	3.57	10	2
C@BC 3	3.56	78.88	3.56	12	2

* U-LCF‒Upper layer incorporated by LCF; C-CFC‒Core layer incorporated by CFC; L-LCF‒Lower layer incorporated by LCF; MUF‒ Melamine-urea–formaldehyde; H‒Hardener.

### Production of biocomposites

**Figure 2:**
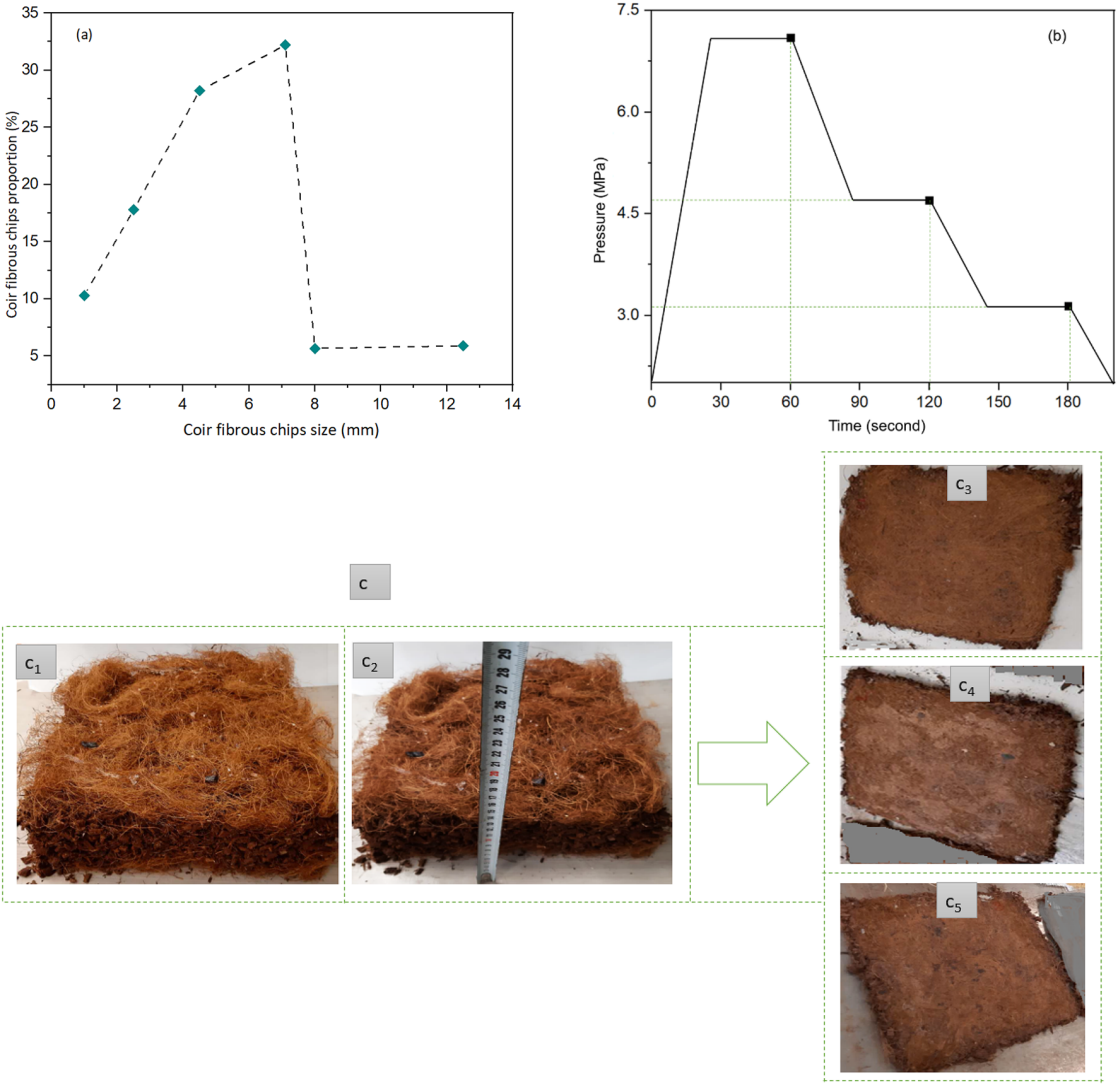
(**a**) Sieve analysis, (**b**) Applied pressure (MPa) against different time intervals for the production of C@BC 1, C@BC 2, and C@BC 3, (**c**) Schematic representation of biocomposites manufacturing process: c_1_ and c_2_: before hot pressing, c_3_, c_4_, and c_5_: after hot pressing.

**Figure 3:**
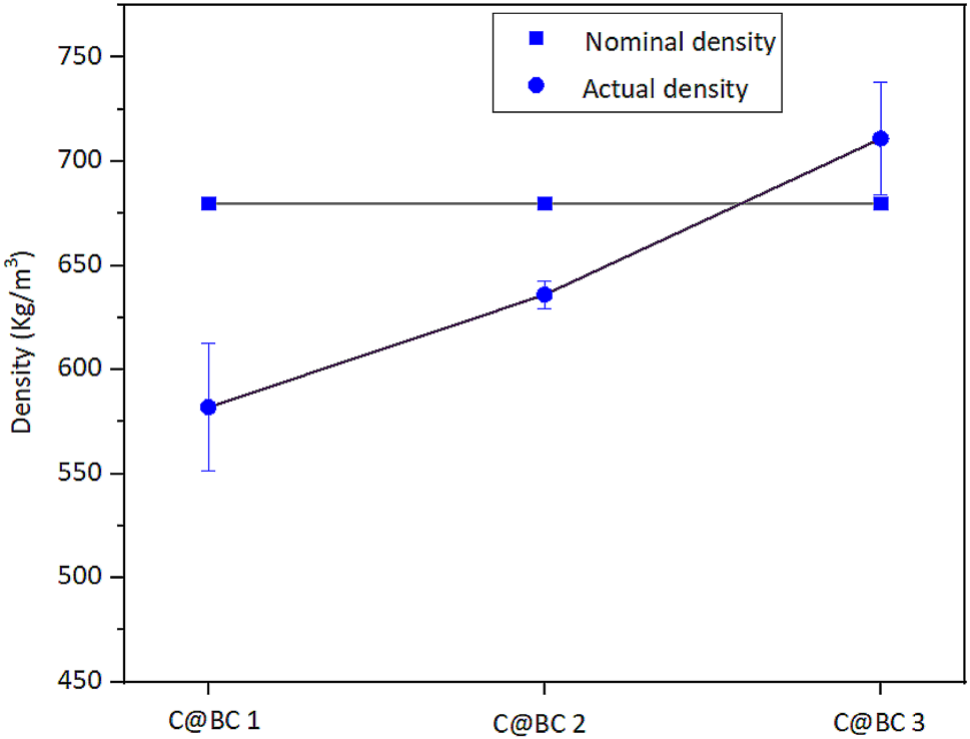
Nominal and actual densities of coir (LCF and CFC) reinforced biocomposites (C@BC 1, C@BC 2, and C@BC 3).

Later on, composite panels were produced by using hot press machine (G. Siempelkamp GmbH and Co. KG. located in Krefeld, Germany). The solution of MUF and hardener were mixed with different ratio as shown in Table 2 at ambient atmospheric (23 °C temperature and 60% relative humidity) condition. The LCF and CFCs were measured as per recipe proportions mentioned in Table 2. The proportions of LCF were 3.58, 3.57, and 3.56%, respectively for C@BC 1, C@BC 2, and C@BC 3 biocomposites both for upper and bottom layers, whereas 82.85, 80.86, and 78.88% CFCs were used as the core layers. Different concentrations of MUF 8%, 10%, and 12% (w/w) and 2% hardeners were used for C@BC 1, C@BC 2, and C@BC 3 biocomposites production. During the calculation of the materials as per recipe, the moisture content of the mat was measured nearly 10‒11%, adhesive 34%, catalyst 65%, and coir materials 7.3%. The CFC was poured in a closed rotating drum and the mixture of MUF and hardeners were sprayed gradually to the coir materials through ensuring uniform mixing. After that, the MUF and hardener mixed coir was transferred to a 300 × 300 × 12 mm^3^ wooden frame over a steel plate and teflon paper with even structural matrix. Three layers were prepared inside the wooden frame (upper layer by LCF, core layer by CFC, and the lower layer again by LCF) as per the recipes mentioned in Table 2. After that, the matrix was covered by using a teflon paper again at upper side too and then another steel plate was placed over it. There were two still rods used to provide 12 mm thickness to the C@BCs. Then, the still plates along with matrix was transferred to the hot press machine. Different pressures 7.1, 4.7, and 3.2 MPa were applied at 180 °C temperature to the composite panels after every 60 s in three stages of pressing (Fig. 2) to release the pressures of steam from the biocomposite panels. The total pressing time was 3 min. After that, the temperature was decreased to room temperature (25 °C) and the pressure was released. Finally, the biocomposite panels were removed from the machine and kept at ambient temperature for cooling. A schematic representation of the produced biocomposite is shown in Fig. 3c. The similar method was followed for composite panel 2 and 3. Thus, three layered coir particleboards were prepared with 300 × 300 × 12 mm^3^ dimensions with different densities (Fig. 3).

### Characterizations of biocomposites (C@BCs)

Thermal conductivity of the biocomposites were measured by using hot plate method as per MSZ EN ISO 10456 2012**.** The measurement requires uniform and flat surface of the composites. All the composite boards were placed in a standard atmospheric condition (65 ± 5% relative humidity and 20 ± 2 °C temperature) for two weeks to achieve the equilibrium moisture content before starting the measurement of thermal conductivity. The difference between the hot and cold side of the plates were 15 °C. After that, all the composite panels were trimmed and cut according to different test requirements and standard by a circular saw cut machine (DCS570N XJ, Pennsylvania, USA). The composite panels were surrounded by 15 cm insulation boards to ensure parallel transfer of heat flow. The measurement of thermal conductivity requires a steady state. So, the measurements were started when last 100 reading were less than 0.002 W/mK. The single measurement was performed in every single minutes. The average of last 100 measurements were considered as the final thermal conductivity. The mechanical properties (tensile, flexural, and internal bonding) of each composite samples (six from each boards) were measured by using Instron 4208 instrument (USA). The density and flexural properties were measured according to EN 310 test standard. The tensile properties were performed as per MSZ EN ISO 527‒4 standard. Test standard (EN 319) was used for the internal bonding strength measurement. The infrared analysis was performed by FTIR instrument (FT/IR-6300, Jasco, Tokyo, Japan). The temperature defined for FTIR study was within the range of 4000‒500 cm^−1^. The microscopic images of the coir and coir-based composites were taken by using the SEM instrument (SEM, S 3400 N, Hitachi, High Technologies Co., Ltd., Tokyo, Japan) within X500 and X1.000 K magnifications at 20.0 kV voltage. The thermal stability of the control and treated coir reinforced composites were carried out by Themys thermal analyzer (TGA, Setaram Instrumentation (Kep Technologies), France). TGA test was performed from 25 °C to 850 °C temperature at 10 °C/min under N_2_ (nitrogen) atmosphere. The thermal degradation reading was taken at onset weight loss after the noteworthy moisture loss.

## Results and discussions

### Density and mechanical properties of coir reinforced biocomposites

Here, three layered biocomposite panels were manufactured with different densities from LCF and CHF coir materials. The apparent densities of the coir (LCF and CHF) reinforced composites were 582 ± 31, 636 ± 7, and 711 ± 27 kg/m^3^. However, the nominal densities were 680 kg/m^3^ (medium density fiberboards). The total density of composites was occupied with the coir (LCF and CFC), correspondent adhesive, and hardeners proportions. The variation in nominal and actual densities (Fig. 2) found maybe for the processing of biocomposites (coir ratio/fiber loading^47^) or with the increased MUF content makes the coir fibers strongly bonded together through reducing void content^48^, hence the C@BC 3 showed higher density comparing to the C@BC 1 and C@BC 2. Besides, the density of fiber composite board is also affected by the porosity and length of the occupying fiber materials^49^. The void content of the produced biocomposites has been calculated using the Eq. (2), where V stands for void content, T_d_ and M_d_ for theoretical and actual density of the biocomposites in Kg/m^3^. T_d_ was calculated according to the weight fractions of coir materials on biocomposite, whereas M_d_ represents measured density. The calculated void contents are 14.41, 6.47, and 4.56%. It is found that void content of the biocomposites increases with the increased volume fractions of fiber, as well as the increased actual density. It maybe that, the increased MUF resin has increased the better bonding between the LCF and CFC materials, thus coir materials and resin materials come into more close contact which minimizes the chance of void creations; thus the mechanical properties also found to be increased (Table 3). Generally, voids are also dependent on processing conditions.

2 $$V = \frac{{T_{d} - M_{d} }}{{T_{d} }} \times 100$$

**Table 3:** Mechanical properties of coir (LCF and CFC) reinforced biocomposites (C@BC 1, C@BC 2, and C@BC 3).

BCs	TS (MPa)	TM (GPa)	FS (MPa)	FM (MPa)	IBS (MPa)	E@B (%)
C@BC 1	3.05 ± 0.45	0.729 ± 268	2.099 ± 0.335	324.0 ± 103	0.15 ± 0.021	4.03
C@BC 2	4.025 ± 0.92	1.379 ± 410	2.659 ± 0.226	937.3 ± 335	0.15 ± 0.036	5.21
C@BC 3	4.400 ± 0.91	1.461 ± 678	5.149 ± 0.148	1775.2 ± 189	0.18 ± 0.07	5.76

BCs, Biocomposites; TS, Tensile strength; TM, Tensile modulus; FS, Flexural strength; FM, Flexural modulus, IBS, Internal bonding strength; E@B, Elongation at break.

**Figure 4:**
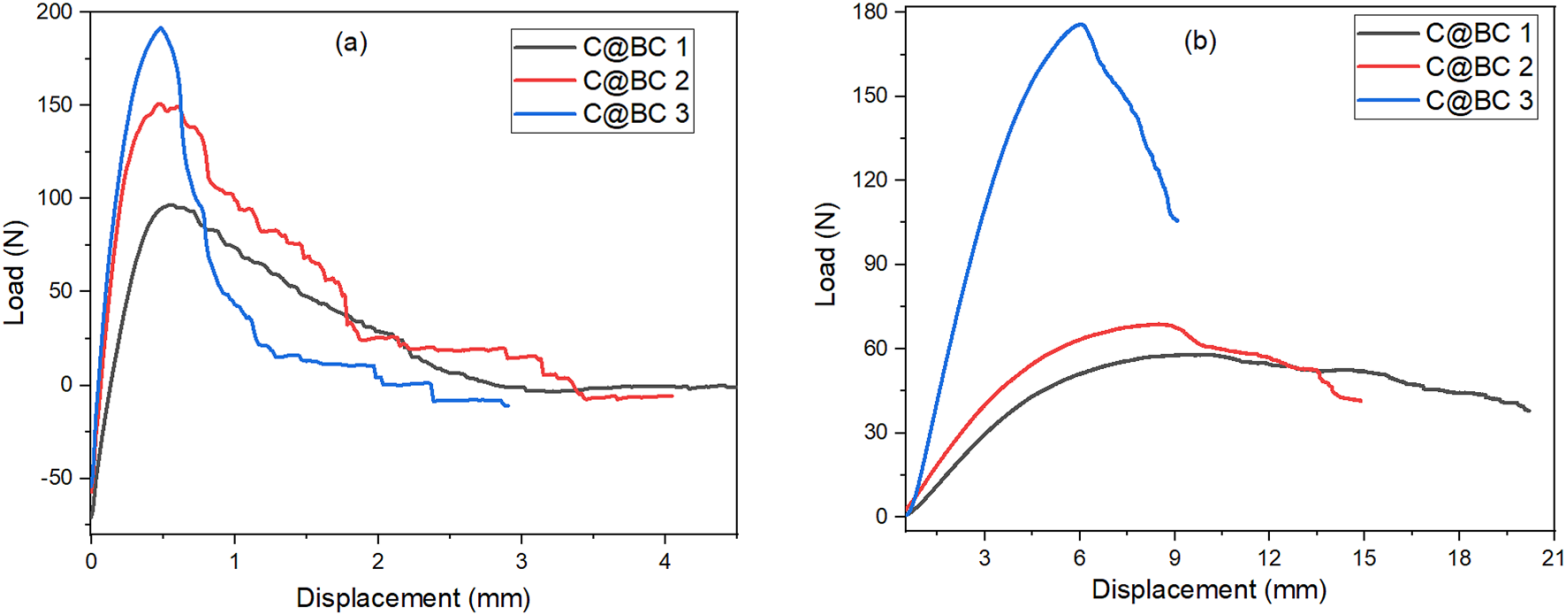
Load versus displacement curves for C@BC1, C@BC2, and C@BC3 composites.

The load and associated displacement is depicted in Fig. 4 both for tensile and flexural tests. The developed biocomposites exhibited a stable growth against the cracking. Generally, the curves showed a rising trends with linear mode initially, although there is a non-linear effect is found when they reach to their highest values of load. The C@BC 1 biocomposite showed the maximum breaking load at 191 N, whereas C@BC 2 and C@BC 3 exhibited the breaking at 150 and 95 N, respectively. However, after showing the highest load, it is started to decrease with the increased delaminations until the total failure of the biocomposites. The similar trends were also observed for flexural displacements as well, where the maximum delaminations were observed at 176, 68, and 58 N loads, respectively for C@BC 1, C@BC 2, and C@BC 3 biocomposites. However, the similar crack propagations were also discussed by other researchers for different natural fibers reinforced thermosetting composites^50–52^.

The mechanical properties of the produced biocomposites (C@BC 1, C@BC 2, and C@BC 3) along with their respective SD (standard deviations) are depicted in Table 2. The tensile strengths varied with the increase of MUF content in all the composites. As only 8% MUF was used for C@BC 1, it provided 3.05 ± 0.45 MPa tensile strength, whereas C@BC 2 provided 4.025 ± 0.92 MPa after using 10% adhesive, and C@BC 3 by 4.400 ± 0.91 MPa for using 12% adhesive. Likewise the tensile strength, C@BC 3 also showed the highest Youngs modulus (1.4614 ± 0.678 GPa), whereas C@BC 2 exhibited the modest, and C@BC 1 showed the lowest performances (Table 3).

The flexural strength and modulus of coir-MUF derived biocomposites are described in Table 3. All the composite types are varied significantly to each other. The values in the table indicates that both the flexural strength and modulus which were increased with the increase of adhesive content. The composite (C@BC 1) showed the lowest flexural strength (2.099 ± 0.335 MPa) and modulus (324.0 ± 103 MPa). Conversely, C@BC 3 exhibited the highest strength (5.149 ± 0.148 MPa) and modulus (1775.2 ± 189 MPa). Although, there is not any report found for multilayered coir with MUF reinforced biocomposites but the composites reported in this study provided better flexural properties compared to one of the recent study of coir reinforced epoxy/polyester composites by Dos Santos et al.^44^. It maybe that, the interfacial adhesion between the pretreated coir and MUF was very good, thus provided better flexural performances. De Olveira et al.^53^ has designed a biocomposite with short coir fiber and epoxy polymer where they have found the flexural strength by 26.7 ± 2.99 to 36.48 ± 2.24 MPa and modulus by 1.41 ± 0.13 to 1.76 ± 0.21 GPa for replicate 1 with variable densities. However, the multilayered biocomposites reported by this study, providing higher bending strength (324.0 ± 103 to 1775.2 ± 189 MPa) and modulus (2.099 ± 0.335 to 5.148 ± 0.148 MPa) for all the panels.

It is found that, biocomposites (C@BC 1 and C@BC 2) showed similar internal bonding strength (0.15) just except the variation in SD. It maybe that, the difference of MUF (2% only) did not effect on their internal bonding significantly. However, when the MUF was increased to 12% there was a significant rise in the internal bonding strength found (0.18 ± 0.07 MPa). Panyakaew and Fotios^54^ has developed binder less coconut husk and bagasse-based insulation fiber boards, where they have found 0.002 MPa internal bonding strength for 350 kg/m^3^ density fiber boards. So, the obtained internal bonding strengths are found reasonable for the medium density fiberboards.

### Morphological properties of coir reinforced biocomposites

**Figure 5:**
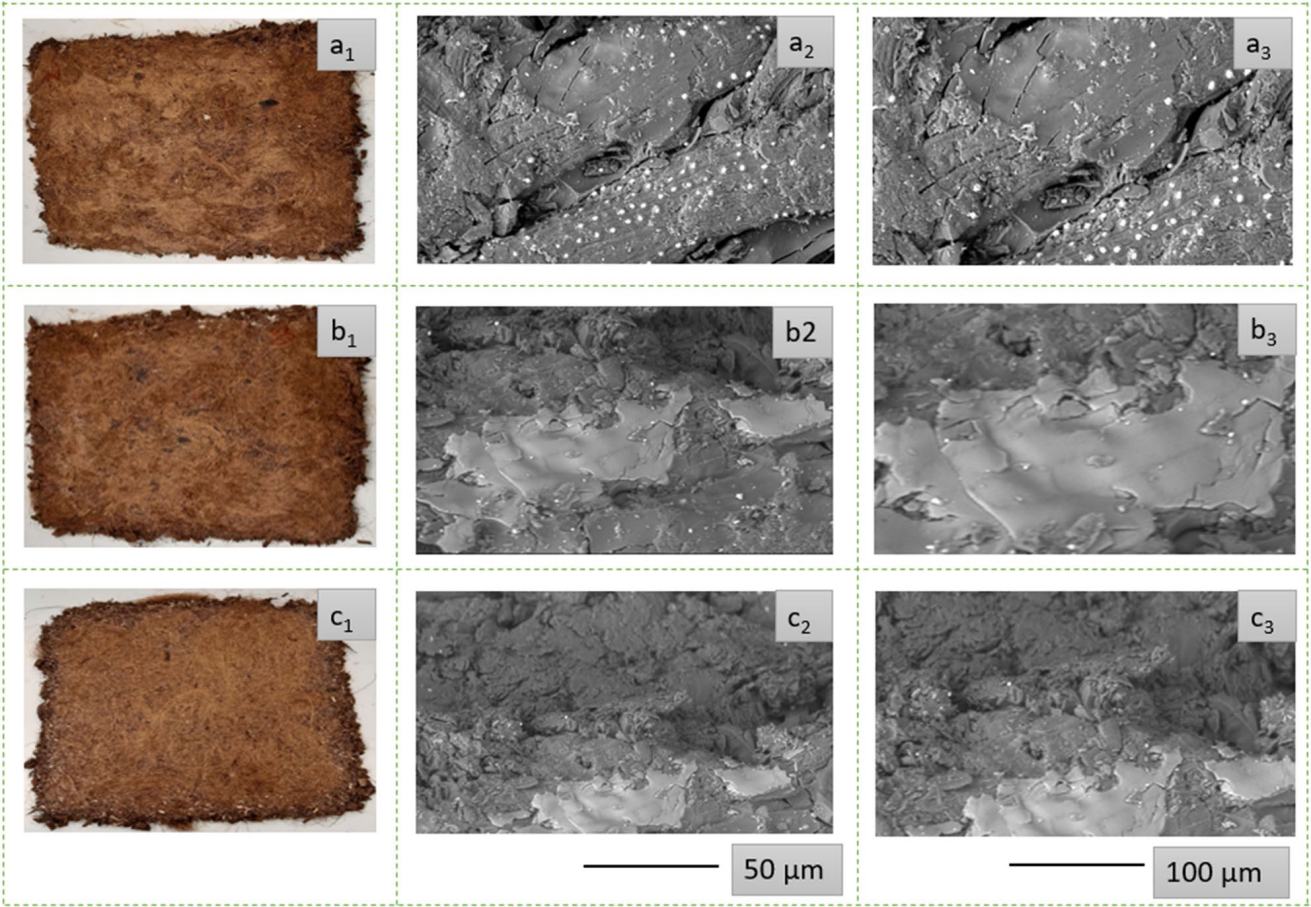
SEM photographs of coir (LCF and CFC) reinforced biocomposites: (a_1_, b_1_, and c_1_) physical photographs of C@BC 1, C@BC 2, and C@BC 3; (a_2_, b_2_, and c_2_) SEM images of C@BC 1, C@BC 2, and C@BC 3 at 50 µm; and (a_3_, b_3_, and c_3_) SEM images of C@BC 1, C@BC 2, and C@BC 3 at 100 µm scale.

The physical and morphological micrographs of produced coir fiber (LCF and CFC) reinforced biocomposites (C@BC 1, C@BC 2, and C@BC 3) are presented in Fig. 5 (a_1_‒c_3_). The surface treatment of LCF and CFC has facilitated to remove the waxy impurities from the fiber surface which consequences for a rougher surface topography. The rougher surface possesses certain competitive advantages to provide better chemical adhesion and bonding between the MUF polymeric resin and coir fiber materials for mechanical interlocking^55^. The photographs exhibit very good adherence between the MUF polymer and coir in the composite systems as seen in Fig. 5 (a_2_, a_3,_ b_2_, b_3_, c_2_, and c_3_). The reason behind the better adherence is the presence of cellulose, lignin, and hemicellulose (Table 1) in the coir fiber components facilitating the interactions into the polar matrix^56^. Besides, the pretreatment of the coir (LCF and CFC) also accelerated the better dispersion of fibers in the polymeric biocomposites^57^. The chemical bonding of the biocomposite panels are seen clearly from the fractured surfaces after applying the tensile loads.

**Figure 6:**
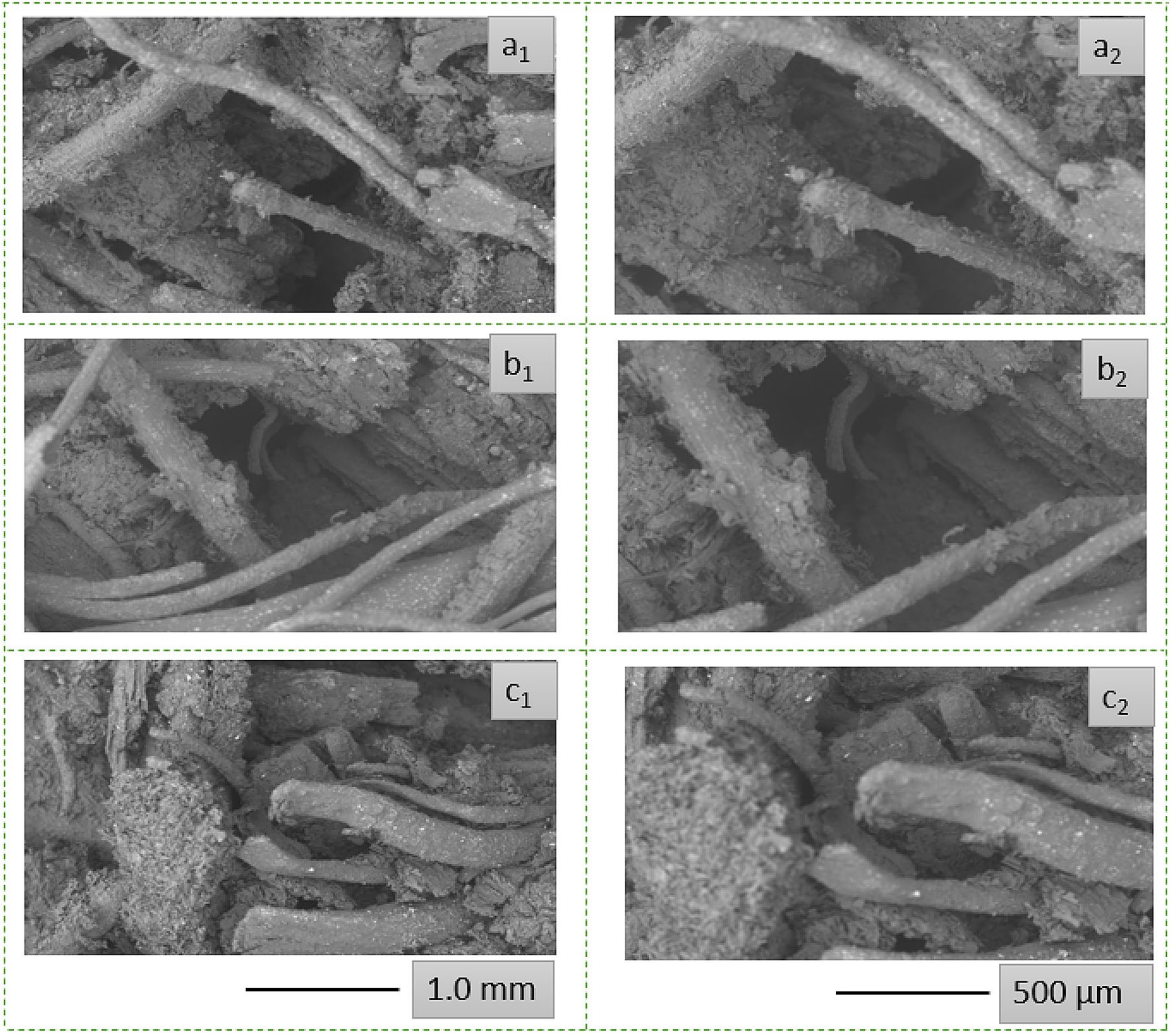
SEM images of fractured coir (LCF and CFC) reinforced biocomposites: a_1_ and a_2_ stands for C@BC 1 biocomposite at 1.0 mm and 500 µm scale, b_1_ and b_2_ stands for C@BC 2 biocomposite at 1.0 mm and 500 µm scale, c_1_ and c_2_ stands for C@BC 3 composite at 1.0 mm and 500 µm scale.

The fractured morphologies of biocomposite panels (C@BC 1, C@BC 2, and C@BC 3) after tensile test is shown in Fig. 6 All the SEM images clearly exhibit the pull-out of coir materials from the biocomposite samples. Although the fibers are not clearly seen in the in Fig. 4 maybe for the strong coating of MUF resin on the biocomposite surface but coir fibers could easily be observed after applying tensile loads (Fig. 6) from all the composite samples. Besides, compared to the morphologies of control coir materials (both LCF and CFC as shown in Fig. 1), the fibers from fractured biocomposite exhibits a rough surface maybe for the better adherence of the MUF resin on pretreated coir^58^. On the other hand, the cracks at coir fiber ends suggest a stronger chemical bonding between the fiber and MUF matrix interface. Likewise, the gap between the matrix and fiber is also seen minimal; which further ensures about the stronger bonding between the polymeric resin and coir fibers^58^. However, the strong chemical bonding also further attributed for the positive effects toward the improved mechanical properties^59^ as reported in Table 3. Furthermore, stronger bonding and adhesion also minimizes the risk of void generation during the biocomposites formation^59^. The overall discussions confirm the successful formations of biocomposites with enhanced thermo-mechanical performances.

### FTIR analysis of coir reinforced biocomposites

**Figure 7:**
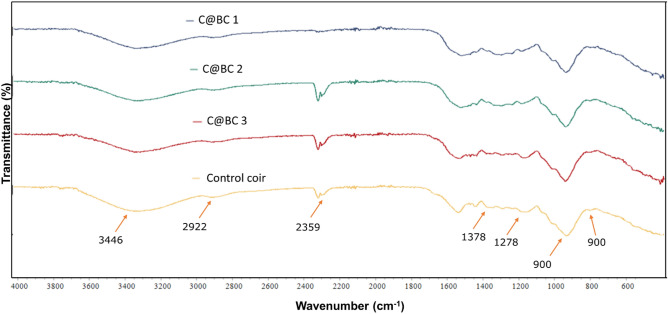
FTIR analysis (4000‒500 cm^‒1^) of control coir, C@BC 1, C@BC 2, and C@BC 3.

The FTIR spectra of pretreated coir, C@BC 1, C@BC 2, and C@BC 3 are shown in Fig. 7. The peaks at 3400 cm^‒1^ is observed for the absorption of O‒H bond which is the common characteristics of coir fibers^60^. The stretched peaks at 2922 cm^‒1^ is representing the symmetric and asymmetric C‒H stretching in the saturated aliphatic component corresponding to the hemicellulose and cellulose^61^. Besides, the bands at 1538 cm^‒1^ also reflects the presence of C = C stretching into the aromatic rings of hemicellulose and lignin^56^. The absorption band at 1378 cm^‒1^ is assigned for the asymmetric and symmetric C‒H deformations in alcohol. The peaks at 1278 cm^‒1^ indicates the deformation and stretching of C‒O bond of cellulose and lignin^61^ (major constituents of coir). The peaks at 900 cm^‒1^ represent the glycosidic bonds of cellulose and hemicellulose^56^. However, there is no major changes appeared after the formation of biocomposites from coir (C@BC 1, C@BC 2, and C@BC 3) which confirms the fingerprints of uniformly distributed coir materials in the matrix system.

### Thermal conductivity of coir reinforced biocomposites

**Figure 8:**
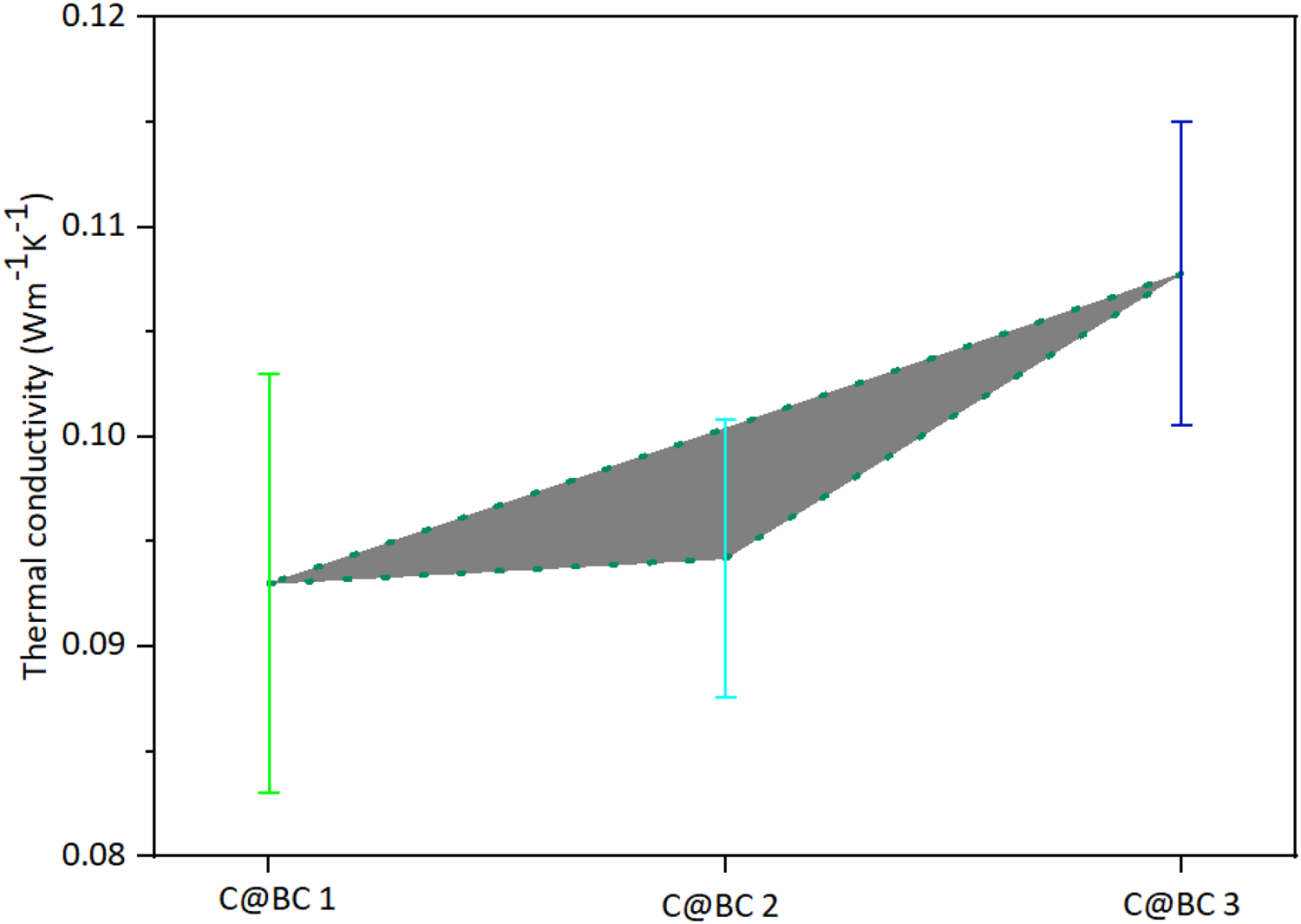
Thermal conductivity of (**a**) control coir, (**b**) C@BC 1, (**b**) C@BC 2, and (**b**) C@BC 3.

The thermal conductivity of the C@BC 1, C@BC 2, and C@BC 3 are provided in Fig. 8. There is a positive relationship between the thermal conductivity and density of the coir fiber boards. The thermal conductivity of the composites is found 0.09302 ± 0.00999, 0.0942 ± 0.0066, and 0.1078 ± 0.0072 W/mK, respectively. It is seen that, the biocomposites showed an increasing trends of thermal conductivity with the increase of densities (Fig. 3). There is no result found for coconut fiber reinforced MUF polymeric composites thermal conductivity previously. However, Panyakew et al.^54^ has reported that composites made without using any adhesive within 250‒350 kg/m^3^ density, provided 0.046‒0.68 W/mK thermal conductivity. As the density of our reported composites are nearly two times higher comparing to that boards^54^, hence the thermal conductivity is also found higher.

### TGA and DTG analysis of coir reinforced biocomposites

**Figure 9:**
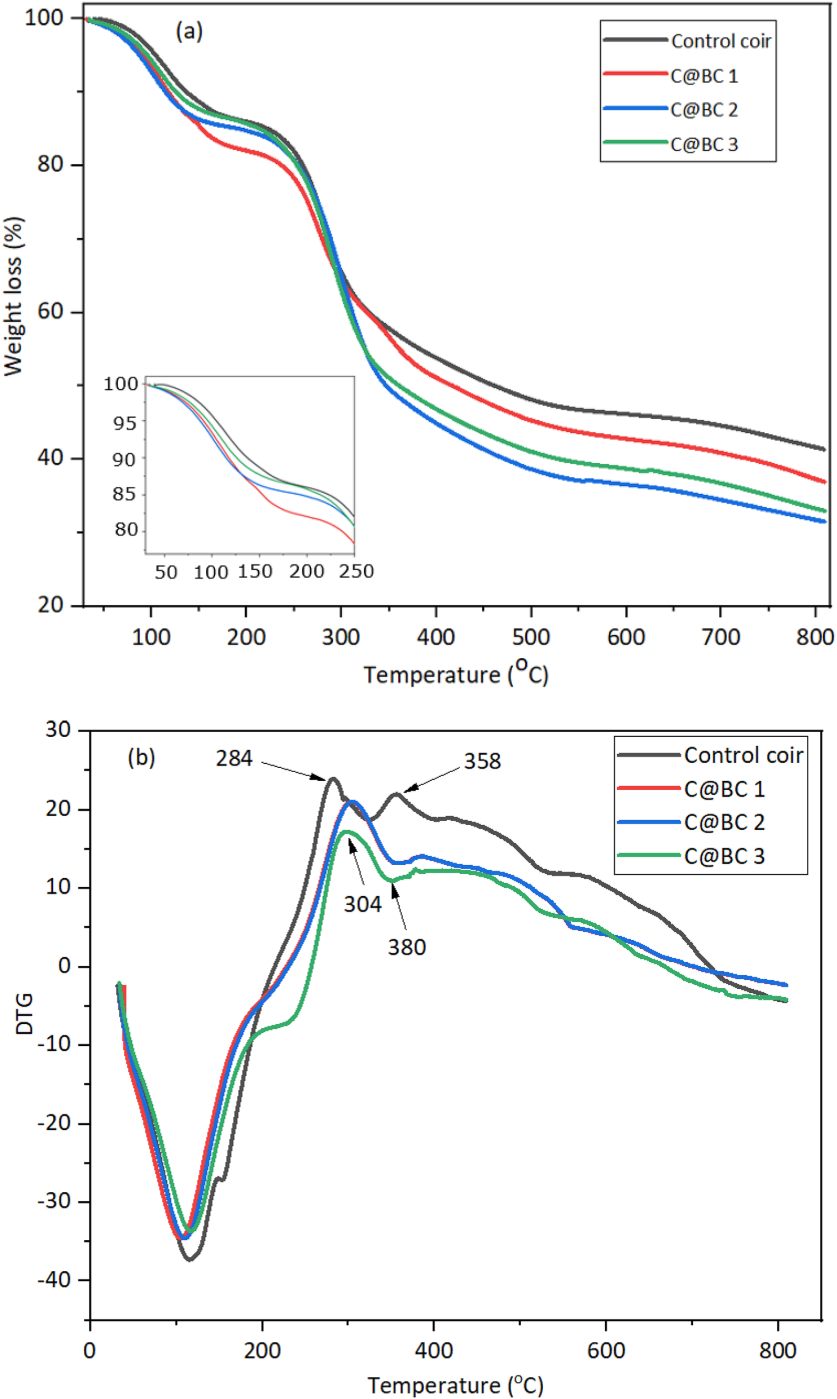
(**a**) TGA and (**b**) DTG analysis of coir (LCF and CFC) reinforced MUF biocomposites.

The thermogravimetric curves of the biocomposites are shown in Fig. 9a,b. Generally, coir exhibits three step thermal degradations^44,60^. The first step degradation is happened for the loss of moisture content due to the vaporization around 100 °C temperature. It is also observed that, control coir showed less weight loss comparing to the composites made from the treated coir. It maybe that alkaline treatment increased the water absorption through removing the impurities and enhancing the higher pores into the coir^56^. The control coir and all the composites showed a significant weight loss at 230‒300 °C maybe for the presence of lower molecular weight of hemicellulose compounds. However, the thermal degradation appeared at 300‒400 °C maybe for the presence of cellulose of coir^60^ and MUF resin. The higher char yield is found maybe for the presence of significant amount of lignin present in coir structure.

However, the DTG curves (Figs. 8b, 9) for the control coir and biocomposites represent the degradation of fibers by maximal kinetics through different peaks. Initially, the peaks for control coir at 284 °C and 304 °C for C@BC 1, C@BC 2, and C@BC 3, respectively correspond the hemicellulose decompositions^44,62^. In this stage, composites made from treated coir showed lower thermal stability compared to untreated/control coir. It maybe that the alkaline treatment has reduced the thermal stability of cellulose^44^. Besides, the peaks at 358 °C for control coir and 380 °C (C@BC 1, C@BC 2, and C@BC 3) are corresponding to the degradations of cellulosic compound^56^. The above mentioned consequences have further confirmed that, the thermo-mechanical treatment of the coir and produced biocomposites has enhanced kinetic decomposition for hemicellulose and cellulose.

### Moisture content of biocomposites

**Figure 10:**
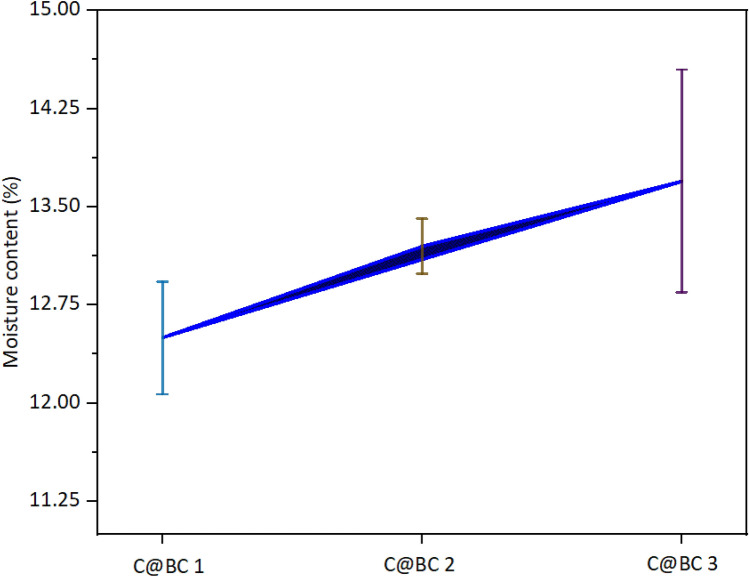
Moisture content of coir (LCF and CFC) reinforced biocomposites.

Moisture content of the produced biocomposite is an important property. The ‒OH group present in the polymeric structure of coir fiber (Fig. 10) material which is responsible for the moisture absorption from surrounding atmosphere^63^. Conversely, the treatment of coir fibers reduces the free ‒OH groups from surface of fibers which consequences for a minimized moisture absorptions^20^. The investigated moisture contents of C@BC 1, C@BC 2, and C@BC 3 are 12.5 ± 0.43%, 13.2 ± 0.21%, and 13.7 ± 0.86%, respectively (Fig. 8). Like as the mechanical properties, moisture content also followed the same trend for the produced biocomposites. But there were no significant differences observed among the biocomposites moisture content%.

## Conclusions

Three multilayered novel biocomposite panels of 582 ± 31, 636 ± 7, and 711 ± 27 kg/m^3^ densities were developed by using coir (LCF and CFC) and MUF adhesive through hot pressing technology at 180 °C temperature for 180 s. The tensile, flexural, and internal bonding strength of board no. 3 (C@BC 3) is comparatively higher than the other two boards (C@BC 1 and C@BC 2). It maybe for the usage of higher MUF% (12) than the other two boards (8% and 10%). The thermal conductivity for all the boards were also found very good ranging from 0.09302 ± 0.00999 to 0.1078 ± 0.0072 W/mK. The thermogravimetric investigation also ensures about the thermal stability of the reported composites. The morphological photographs reflected excellent adhesion property between the MUF polymer and coir in the matrix system. The FTIR analysis provided the footprint of structural coir in composite panels. The overall investigations have proven the successful formations of biocomposite panels with improved thermo-mechanical performances which could be feasible for industrial applications.
